# Real-time realizable mobile imaging photoplethysmography

**DOI:** 10.1038/s41598-022-11265-x

**Published:** 2022-05-03

**Authors:** Hooseok Lee, Hoon Ko, Heewon Chung, Yunyoung Nam, Sangjin Hong, Jinseok Lee

**Affiliations:** 1 Department of Biomedical Engineering, Kyung Hee University, Yongin, Republic of Korea; 2 Department of Computer Science and Engineering, Soonchunhyang University, Asan, Republic of Korea; 3 Department of Electrical Engineering, SUNY-Stony Brook University, Stony Brook, NY USA

**Keywords:** Health care, Engineering

## Abstract

Photoplethysmography imaging (PPGI) sensors have attracted a significant amount of attention as they enable the remote monitoring of heart rates (HRs) and thus do not require any additional devices to be worn on fingers or wrists. In this study, we mounted PPGI sensors on a robot for active and autonomous HR (R-AAH) estimation. We proposed an algorithm that provides accurate HR estimation, which can be performed in real time using vision and robot manipulation algorithms. By simplifying the extraction of facial skin images using saturation (S) values in the HSV color space, and selecting pixels based on the most frequent S value within the face image, we achieved a reliable HR assessment. The results of the proposed algorithm using the R-AAH method were evaluated by rigorous comparison with the results of existing algorithms on the UBFC-RPPG dataset (*n* = 42). The proposed algorithm yielded an average absolute error (AAE) of 0.71 beats per minute (bpm). The developed algorithm is simple, with a processing time of less than 1 s (275 ms for an 8-s window). The algorithm was further validated on our own dataset (BAMI-RPPG dataset [*n* = 14]) with an AAE of 0.82 bpm.

## Introduction

Owing to their capacity to measure heart rates (HRs) without any contact with human skin, photoplethysmography imaging (PPGI) sensors have been the focus of considerable attention. A PPGI sensor uses a camera with the capability of face detection and records images of facial skin, as skin can represent changes in arterial blood volume between the systolic and diastolic phases of the cardiac cycle^1,2^. Thus, these sensors enable remote monitoring of HRs and do not require any device to be worn on the finger^3–5^ or wrist^6–8^. However, these convenient monitoring capabilities have not led to successful commercialization through FDA approval, because they have a lower accuracy than contact-PPGs, as they can be sensitive to human movement and ambient light change. Recently, PPGI sensors have been utilized in webcams (as a low-cost and low-quality solution) or high-quality industrial cameras (as a high-cost and better-quality solution) connected to a computer; moreover, various studies have presented diverse algorithms that have yielded high accuracy results^9–17^. However, this is a static approach, which makes it difficult to measure HRs during daily life activities; moreover, it makes practical application difficult in many real-life medical fields. In addition, most studies do not consider the computational complexity because a general purpose computer connected to a camera deals with the entire required process including face detection, face skin extraction, PPG acquisition and HR estimation. We believe that, for PPGI sensors to be applied and utilized in various medical fields, it is necessary to acquire PPG signals without space restrictions.

To extend the use of PPGI sensors, in this paper, we propose an algorithm that provides accurate HR estimation, and can be performed in real time using vision and robot manipulation algorithms. We mounted PPGI sensors on a robot for active and autonomous HR (R-AAH) estimation. This dynamic approach allowed the robot to actively monitor HRs, which enables active medical services; the services include providing HR information to people in the vicinity of the robot. The proposed R-AAH navigates a specific physical space, while avoiding obstacles. It recognizes human faces and records images of facial skin while in motion; these images are then converted into PPGI signals, which are used in the estimation of the person's HR. More specifically, R-AAH involves six stages: simultaneous localization and mapping (SLAM), robot navigation, face detection, facial skin extraction, PPGI signal conversion, and HR estimation. SLAM is the initial stage, whereby the robot constructs or updates a map of an unknown environment while simultaneously keeping track of its position^18^. During the robot navigation stage, the robot determines its own spatial position and constructs a plan for a path toward a designated position^19^. The face detection stage uses computer vision to detect faces in the environment^20^. The facial skin extraction stage identifies facial skin pixels that change according to the cardiac cycle. The conversion of the PPGI signal and estimation of the person's HR are then used to compute the HR value using the acquisition of PPG signals obtained from the facial skin pixels over time.

For the fast conversion of the PPG signal and estimation of the HR, we propose a simplified facial skin extraction algorithm, which allows for accurate HR estimation in real-time, even when using low-power hardware. We focused on reducing the computational complexity of the algorithm and increasing the accuracy for real-time implementation. Notably, the aim of this study is not to obtain a full-fledged optimized robot system, but to describe the implementation of all the six required stages, and focus on the development of an achievable real-time system for estimating HR values per second using low-power hardware. In particular, we optimize the facial skin image by selecting pixels based on the most frequent saturation (S) value in the image, which resolved one of the obstacles of real-time operation.

## Methods

### Robot system description

**Figure 1:**
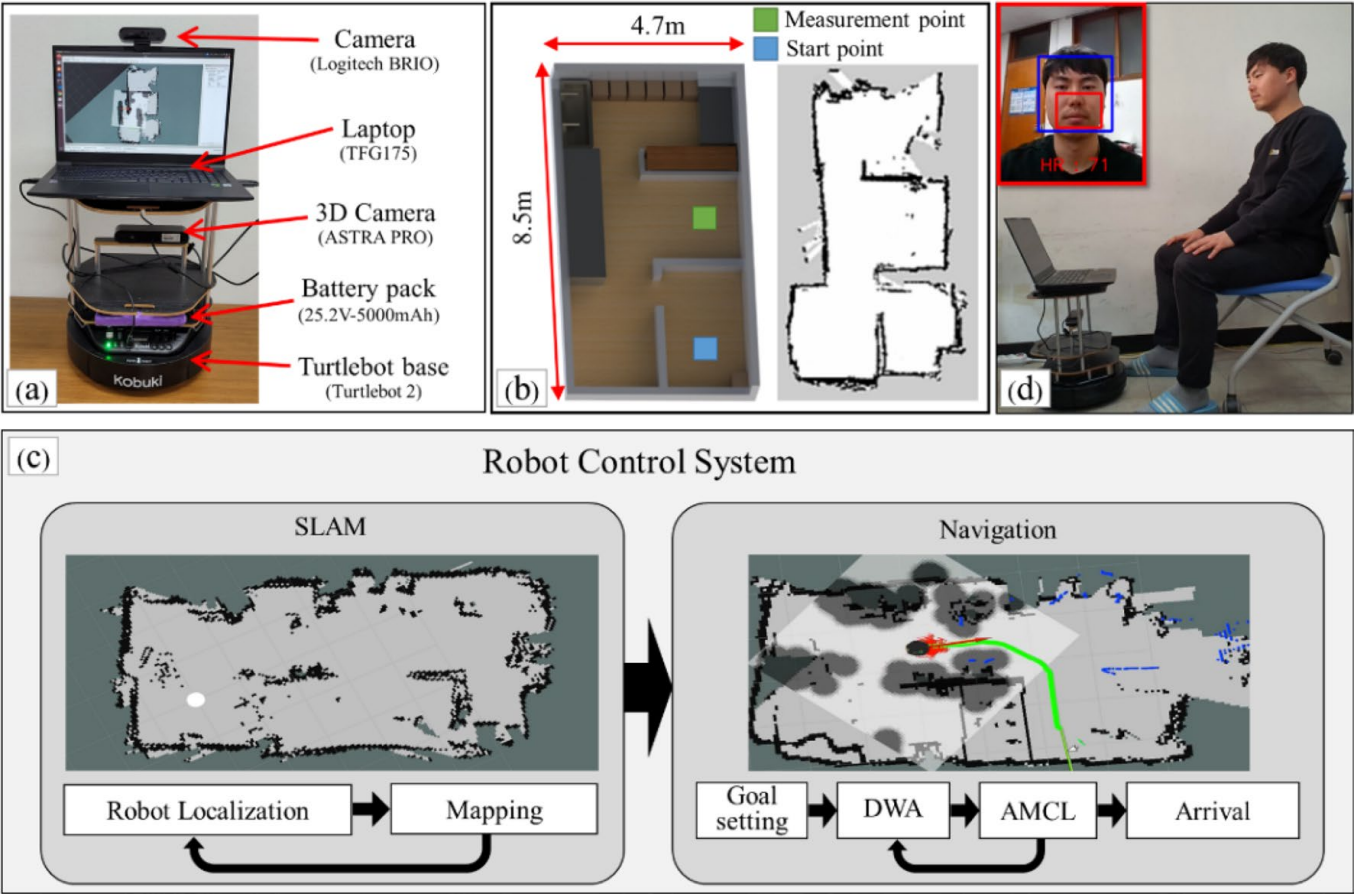
Overview of the proposed system for HR estimation using a PPGI mounted on a robot; (**a**) Robotic device based on a turtlebot2 framework with a 3D camera, a laptop and a webcam; (**b**) SLAM and navigation; (**c**) Real-time HR estimation via face detection, face skin image extraction, PPGI acquisition; (**d**) Algorithm flow chart for SLAM and navigation.

Figure 1 shows our designed device for active and autonomous HR estimation using a robot equipped with PPGI sensors. The complete system involves six stages, as outlined in the previous section. Figure 1a shows the robot we developed using a turtlebot2 framework (YUJIN ROBOT, Incheon, Korea). Within the framework, a three-dimensional (3D) camera (ASTRA PRO, ORBBEC, Michigan, USA) was mounted on the device for the SLAM and robot navigation stages; a web camera (Logitech BRIO, Switzerland) was used for the remaining stages. Both cameras were operated through a laptop computer (TFG175, Hansung, Seoul, Republic of Korea) with an AMD Ryzen 5 3400G, 3.70 GHz processor (having 8 GB RAM with 8 threads). The web camera had a frame rate of 30 frames per second (fps) and a pixel resolution of 640 × 480 in an uncompressed 8-bit RGB format.

The device was provided with an initial map that indicated the designated start and destination coordinates (Fig. 1b, left); the robot then performed the SLAM, constructed a map of its surroundings based on data from the 3D camera (Fig. 1b, right), and localized its position within the mapped environment^18,21^. SLAM iterates the mapping and localization data (Fig. 1c, left) with respect to the initial map. In this study, we used a factored solution, FastSLAM, which estimated the robot’s position using a particle filter, and updated the map using an extended Kalman filter^22^.

Once the robot completed the SLAM, it navigated to the designated start position, and continued until it reached the designated destination. The navigation stage included two iterative steps of localization and pathfinding (Fig. 1c, right); we used the adaptive Monte Carlo localization (AMCL), known as particle filter localization^23–25^, for localization. The algorithm used a particle filter to determine the distribution of likely states to define where the robot was initially localized, combined with a posterior particle density estimation function. With each movement of the robot, the device updated the particle distribution to predict its new state (position and velocity); then, the particles were resampled using recursive Bayesian estimation based on measurements obtained (depth information) from a 3D camera. For the path-finding step, we used a dynamic window approach (DWA) to efficiently generate the trajectory of subsequent movement^26^. A DWA is an online collision avoidance strategy for mobile robots, derived directly from the dynamics of the robot; it is normally designed to adapt to the constraints imposed by the limited velocities and accelerations of the robot.

As the robot navigated, it searched for a human in its surroundings by detecting faces using the web camera. For real-time face detection, we used a deep neural network (DNN) based single shot scale-invariant face detector (S3FD)^27^. The S3FD uses a scale-equitable framework with a wide range of anchor-associated layers and a series of appropriate anchor scales to handle different facial sizes. The architecture consists of a truncated VGG-16 network with extra convolutional layers, detection convolutional layers, normalization layers, predicted convolutional layers, and a multi-task loss layer. The detection layers are associated with specific anchor scales, ranging from 16 to 512, which enable the robot to detect different facial sizes. The DNN was trained using 12,880 images from the WIDER FACE training set^28^, and the trained model achieved state-of-the-art performance on the majority of common face detection benchmarks, such as Annotated Faces in the Wild (AFW)^29^, PASCAL Face^30^, Face Detection Dataset, and the Benchmark (FDDB)^31^. The results of the face detection are summarized in Supplementary Table 1, where the WIDER FACE dataset was used for evaluation, and S3FD was used for face detection in this study.

Once a face was detected, the robot paused its navigation and performed HR estimation using PPG acquisition. The image with the detected face was then categorized into two regions: facial skin providing pulsatile information, and non-facial skin, such as background or hair. The face skin regions were extracted from the image, and the non-pulsatile information was removed. Finally, the images of the extracted regions were converted into a PPGI signal, which provided a real-time HR value (Fig. 1d). The robot estimated the person's HR for 1 min, after which it resumed navigation to the original destination, repeating its search for a human in its surrounding. The process was repeated until the robot reached the final destination.

### Problems with real-time PPG acquisition

In our proposed system, video images are acquired at a rate of 30 frames per second (fps). A key aspect of this study is to ensure that all steps, including those of face detection, skin image extraction, PPG signal conversion, and HR estimation, are realizable in real-time. Thus, for the given frame rate, to acquire a 1 s PPG, we need to perform 30 face detections and 30 skin image extractions from 30 video images within a second. A short PPG window can increase time-resolution, while a long PPG window can improve SNR. However, the gain in performance from using a longer window comes at the price of an increased latency. In this study, for real-time HR estimation, we used an 8 s window PPGI segment per second (i.e., an 8 s window with a 1 s shift), similar to the parameters used in previously reported algorithms^8,32^. Using this method, we can provide an 8 s average HR value every second. However, the following question arises: is it possible to acquire the PPG signal and compute HR measurements within a second on a single CPU? Most previous studies have focused on the accuracy of HR estimation using PPGI^17,33–36^; however, such an algorithm, when applied to a robot in a system similar to the one we designed in this study, should focus not only on accuracy, but also on the complexity of the computations. Although it is beyond the scope of the current study, via accurate and real-time HR estimation, a robot can perform additional real-time calculations such as heart rate variability (HRV) analysis^37^, atrial fibrillation diagnosis^38^, and cardiac rehabilitation^39,40^, based on HR values.

### Overall data flow

**Figure 2:**
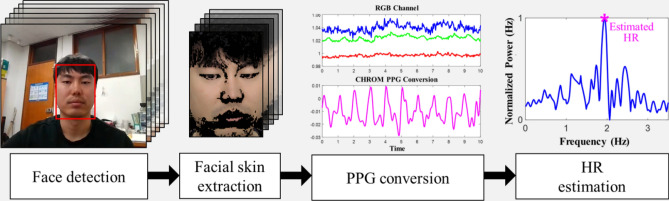
Overview of the data flow for HR estimation.

Figure 2 illustrates the data flow for the estimation of a person's HR. First, faces are detected in consecutive image frames (240 frames in 8 s, given the frame rate of 30 fps) and facial skin regions are extracted. The extracted images of multiple skin regions are then converted to PPG. Finally, HR is estimated using power spectrum analysis.

### Face skin extraction with relative saturation value range (RSVR)

A facial landmark-based approach has been widely described for the task of extracting facial skin from an image^17,33,34,41^. This approach involves the recognition of the geometric structure of faces in images, and obtains a canonical alignment of a face based on translation, scale, and rotation. The resulting facial landmark-based networks proposed a variety of facial skin areas, such as rectangle-, bottom faced-, and polygon-shaped areas; they were able to estimate HRs with high accuracy. However, the use of the face landmark-based approach lead to heavy computational complexity. This can make real-time processing difficult when computing HR values per second or when the robot is connected to a personal laptop computer.

**Figure 3:**
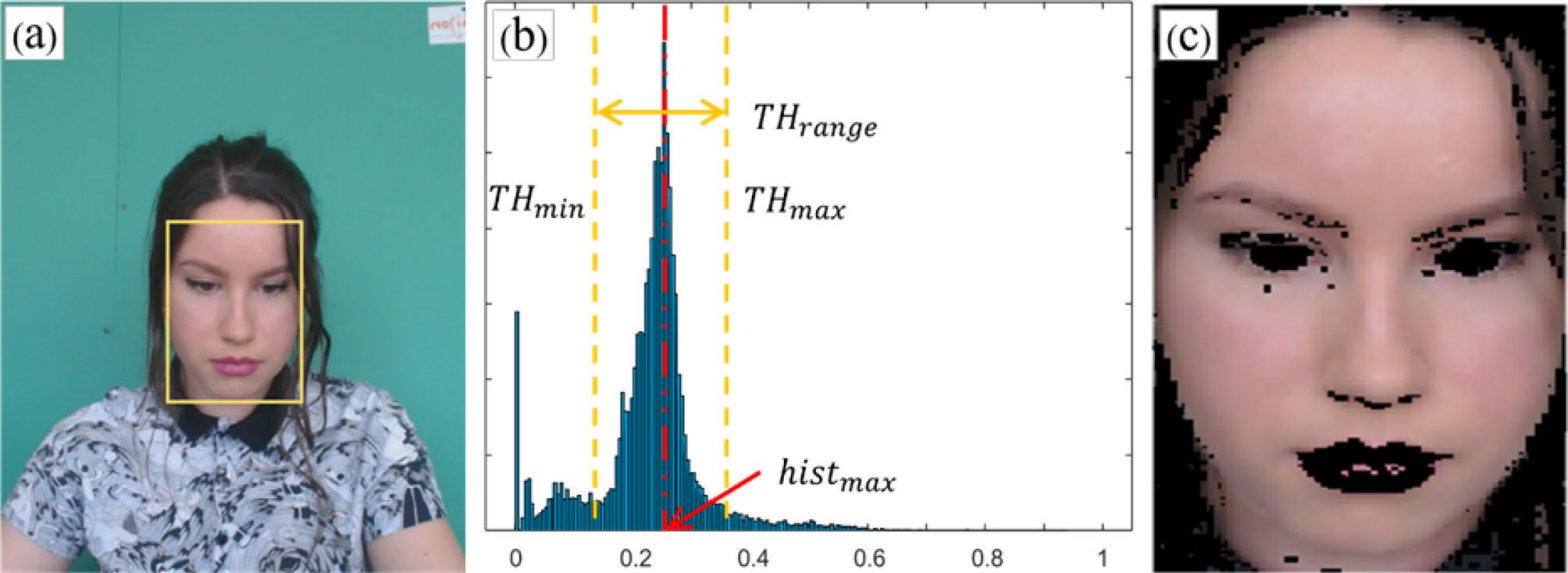
Overview of face skin extraction (from the public dataset UBFC-rPPG): (**a**) detected face is outlined by a yellow rectangle and may include hairs and parts of the background; (**b**) histogram of S values, distributed around the center value $hist_{max}$ (the most frequent S value on the face image); (**c**) resultant face skin image after applying the relative saturation (S) value range.

To reduce the computational complexity, we identified facial skin areas using S values in the HSV color space. However, the detected face image may sometimes include hair and/or background that is not facial skin (Fig. 3a, yellow rectangle). Because the hair and background images do not contain any pulsatile information, the face skin extraction stage is one of the most important stages in acquiring a clean PPG signal. To extract only the facial skin areas (pixels) as the region of interest (ROI), we first converted the images with a detected face (inside the rectangle area) to HSV color space images, and obtained a histogram of the converted S values. We then applied a median filter of length 5 to the histogram (Fig. 3b). The most frequent S value in the image was denoted by $hist_{max}$, and the width of the considered face skin region centered at $hist_{max}$ was defined as $TH_{range}$. The S value in the (i, j) pixel from the k-th frame image was denoted by $S_{k}^{ij}$, and satisfies the following condition:

1 $$ hist_{max} - TH_{range} /2 < S_{k}^{ij} < hist_{max} + TH_{range} /2. $$

The red, green, and blue values in the (i, j) pixel from the original image in the k-th frame were denoted by $R_{k}^{ij}$, $G_{k}^{ij}$, and $B_{k}^{ij}$, respectively, and the red, green, and blue values in corresponding pixels in the face skin image at the $k$-th frame were denoted by $R\left( f \right)_{k}^{ij}$, $G\left( f \right)_{k}^{ij}$ and $B\left( f \right)_{k}^{ij}$, respectively. Then, $R_{k}^{ij}$, $G_{k}^{ij}$ and $B_{k}^{ij}$ can be expressed as:

2 $$ \left[ {\begin{array}{*{20}c} {R\left( f \right)_{k}^{ij} } \\ {G\left( f \right)_{k}^{ij} } \\ {B\left( f \right)_{k}^{ij} } \\ \end{array} } \right] = \left\{ {\begin{array}{*{20}c} {\left[ {\begin{array}{*{20}c} {R_{k}^{ij} } & {G_{k}^{ij} } & {B_{k}^{ij} } \\ \end{array} } \right]^{T} ,} & {\quad \left\langle {\begin{array}{*{20}c} {hist_{max} - TH_{range} /2 < S_{k}^{ij} } \\ { < hist_{max} + TH_{range} /2} \\ \end{array} } \right.} \\ {\left[ {\begin{array}{*{20}c} 0 & 0 & 0 \\ \end{array} } \right]^{T} ,} & {\quad otherwise} \\ \end{array} } \right., $$

To find the optimum value of $TH_{range}$, we set the value to be proportional to the size of the value $hist_{max}$, as follows:

$$ TH_{range} = \alpha \cdot hist_{max} $$

where α is a constant. In this study, we set $\alpha = 0.2$. We investigated the effects of a constant value α, and the method of choosing the S feature over the hue (H), value (V), red (R), green (G), and blue (B) to extract the face skin regions; these investigations are discussed in the “Results” section. Figure 3c shows the resultant face skin image obtained. Recently, Boccignone et al. argued that facial skin extraction requires an adaptive threshold technique because each face has its own features^42^. However, in this study, because the relative S value range (RSVR) extracts the face skin pixels based on the different $hist_{max}$ values in each image, the resultant successive image pixels over time are able to represent the pulsatile component of the cardiac cycle under different conditions (i.e., ambient light and/or different subjects). Furthermore, the RSVR-based facial skin extraction method significantly reduces the complexity of the computations compared to current state-of-the-art methods^17,33,34,41^.

### PPG conversion and HR estimation

Given the face skin image of $$\left[ {\begin{array}{*{20}c} {R\left( f \right)_{k}^{ij} } & {G\left( f \right)_{k}^{ij} } & {B\left( f \right)_{k}^{ij} } \\ \end{array} } \right]^{T}$$, all pixels corresponding to facial skin were averaged for each of the three channels as

3 $$ \left[ {\begin{array}{*{20}c} {\overline{R}\left( f \right)_{k} } \\ {\overline{G}\left( f \right)_{k} } \\ {\overline{B}\left( f \right)_{k} } \\ \end{array} } \right] = \left[ {\frac{1}{{W \cdot H - C_{k}^{otherwise} }} \cdot \mathop \sum \limits_{i = 1}^{W} \mathop \sum \limits_{j = 1}^{H} \left[ {\begin{array}{*{20}c} {R\left( f \right)_{k}^{ij} } \\ {G\left( f \right)_{k}^{ij} } \\ {B\left( f \right)_{k}^{ij} } \\ \end{array} } \right]} \right] $$

where $\overline{R}\left( f \right)_{k}$, $\overline{G}\left( f \right)_{k}$ and $\overline{B}\left( f \right)_{k}$ represent the averaged pixel value for each channel, $W$ and $H$ are the width and height of the detected face image (rectangle area), and $C_{k}^{otherwise}$ is the number of pixels in the rectangle area that are outside the optimal range $hist_{range}$ for each channel. The averaged pixel values for each channel can be arranged according to the image frame as

4 $$ \begin{array}{*{20}c} {R_{1:N} = \left[ {\begin{array}{*{20}c} {\overline{R}\left( f \right)_{1} ,\overline{R}\left( f \right)_{2} } & \cdots & {\overline{R}\left( f \right)_{N} } \\ \end{array} } \right]} \\ {G_{1:N} = \left[ {\begin{array}{*{20}c} {\overline{G}\left( f \right)_{1} ,\overline{G}\left( f \right)_{2} } & \cdots & {\overline{G}\left( f \right)_{N} } \\ \end{array} } \right]} \\ {B_{1:N} = \left[ {\begin{array}{*{20}c} {\overline{B}\left( f \right)_{1} ,\overline{B}\left( f \right)_{2} } & \cdots & {\overline{B}\left( f \right)_{N} } \\ \end{array} } \right]} \\ \end{array} $$

where N is the total number of image frames. Hence, the 8 s red channel data corresponding to the 240 samples can be expressed as

5 $$ R_{s + 1:s + 240} = \left[ {\begin{array}{*{20}c} {\overline{R}\left( f \right)_{s + 1} ,\overline{R}\left( f \right)_{s + 2} } & \cdots & {\overline{R}\left( f \right)_{s + 240} } \\ \end{array} } \right] $$

where $s + 1$ represents the starting image frame. Note that green and blue channel data can be expressed similarly (i.e. $G_{s + 1:s + 240} \;{\text{and}}\;B_{s + 1:s + 240}$). Based on $R_{s + 1:s + 240}$, $G_{s + 1:s + 240}$, and $B_{s + 1:s + 240}$, we applied the chrominance-based (CHROM)^37^, and derived the PPG signal, $S_{s + 1:s + 240}$, as follows:

6 $$ X_{s + 1:s + 240} = 3R_{s + 1:s + 240} - 2G_{s + 1:s + 240} $$

7 $$ Y_{s + 1:s + 240} = 1.5R_{s + 1:s + 240} + G_{s + 1:s + 240} - 1.5B_{s + 1:s + 240} $$

8 $$ S_{s + 1:s + 240} = X_{s + 1:s + 240} - \beta Y_{s + 1:s + 240} $$

where

9 $$ \beta = \frac{{\sigma \left( {X_{s + 1:s + 240} } \right)}}{{\sigma \left( {Y_{s + 1:s + 240} } \right)}} $$

where the operator σ is the standard deviation.

Using this approach, we were able to obtain an 8 s PPG window signal every second. For each window signal, we applied a fourth-order Butterworth bandpass filter (BPF), with cutoff frequencies of 0.4 and 4 Hz. All subjects had HRs in the approximate range of 40–200 bpm, which includes both at rest subjects and subjects engaging in a high-intensity physical activity^43–45^. The filtered signal was then normalized to a zero mean with a unit variance. Subsequently, we estimated the power spectral density (PSD) of the filtered signal using Welch's method^46^, in which the segment was divided into 8 sub-segments with 50% overlap, and each sub-segment was windowed with a Hamming window. Finally, we found the maximum power frequency $f_{HR}$ (Hz), and estimated the HR as $HR_{est} \left( t \right) = 60 \cdot f_{HR}$ bpm. The aforementioned framework for HR estimation using RSVR is summarized in Algorithm 1.
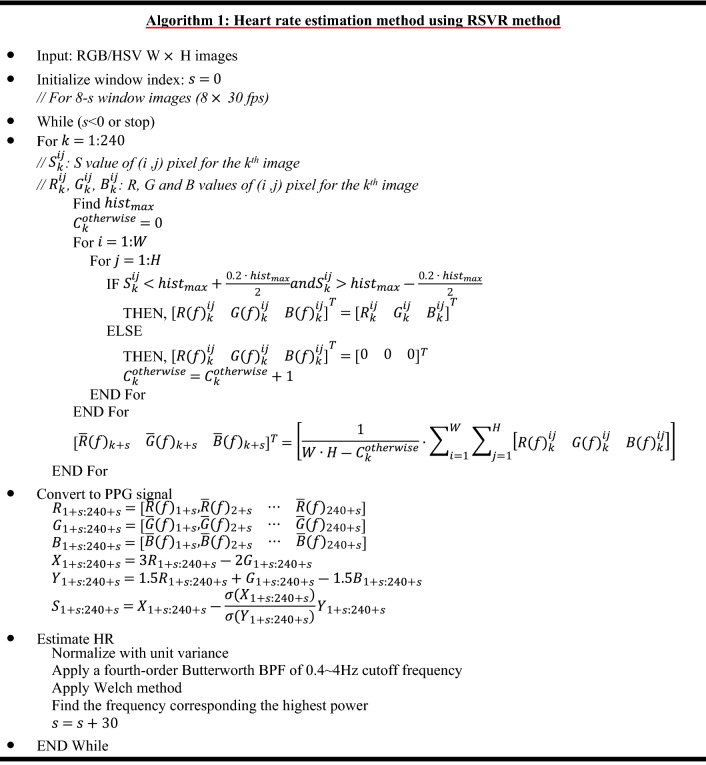


### UBFC-rPPG dataset

The publicly released UBFC-RPPG^47^ dataset was used for training; this dataset is specifically designed for remote HR measurement tasks, and contains 42 one-minute long videos from 42 different subjects. All participants provided consent for the publication of identifying images in an online open-access publication. The videos were recorded using a Logitech C920HD Pro camera with 30 fps and a resolution of 640 × 480 pixels in an uncompressed 8-bit RGB format. Each subject was made to sit in front of a camera at a distance of approximately 1 m. Subjects were required to play a time-sensitive mathematical game, which caused variations in their HRs. The video recorded the natural rigid and non-rigid movements of the subjects. During the video recording, a transmitting pulse oximeter CMS50E-based PPG signal was simultaneously measured from a finger to obtain a reference HR, denoted $HR_{true} \left( t \right)$. Pulse peaks in the reference HR were identified and inter-beat intervals were calculated, which were then resampled to 4 Hz by fitting a cubic spline to obtain continuous HR values.

### BAMI-rPPG dataset

We tested our proposed algorithm, with the R-AAH framework, in real time. This testing dataset, named BAMI-rPPG, comprised a total of 14 participants (10 male and 4 female), with an average age of 29.21 ± 2.36 years. This study was approved by the institutional review board of Wonkwang University in Korea. All participants provided written informed consent. All methods were performed in accordance with the relevant guidelines and regulations. The BAMI-rPPG dataset was built using our designed robot navigation system, as outlined in Fig. 1. Each participant was randomly positioned in an indoor environment, measuring 8.5 m × 4.7 m, which the robot navigated using SLAM, searching for a human using face detection. When a face was detected, the face images were recorded, the facial skin area was extracted with the relative S value range, and the CHROM method was used for PPG acquisition. Every second, the final 8-s PPG was filtered, and the HR was estimated using PSD. During the 1 min HR estimation, a transmitted PPG signal was obtained from the finger-type oxygen saturation device for the reference HR, $HR_{true} \left( t \right)$. As for the UBFC-rPPG dataset, we first found the pulse peaks of the reference HR and then calculated the inter-beat intervals, which were resampled to 4 Hz by fitting a cubic spline to obtain continuous HR values.

### Evaluation and metrics

**Figure 4:**
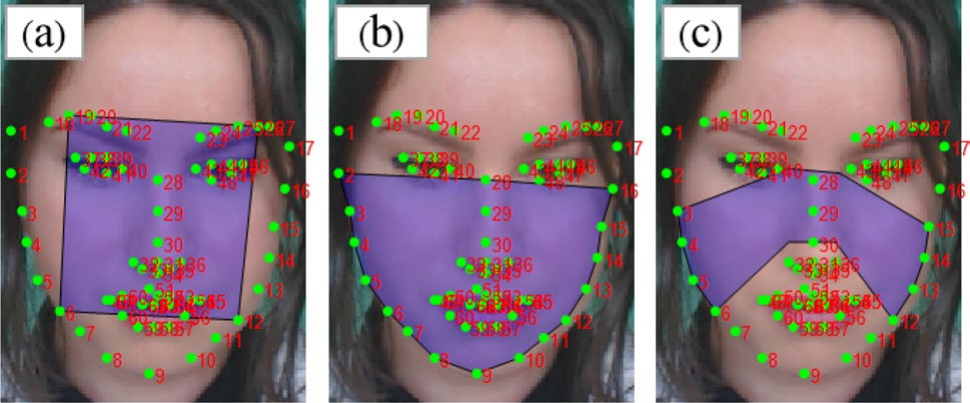
Landmark-based face skin extraction: (**a**) a rectangular face ROI^17^, (**b**) a bottom face ROI^33^, and (**c**) a polygonal face ROI^34^.

Python 3.6.8, OpenCV 4.2.0, NumPy 1.18.2, SciPy 1.4.1, Scikit-learn 0.22.2, TensorFlow 1.8.0 and ROS Melodic 1.14.5, including SLAM, navigation and Turtlebot packages, were used for the implementation of our proposed algorithm and R-AAH method. We investigated the effect of selecting the S feature over the hue (H), value (V), red (R), green (G), and blue (B) values for facial skin extraction in the training dataset, UBFC-RPPG (n = 42). In addition, we investigated the effects of the parameter α by varying it from 0.1 to 0.5, at intervals of 0.1. Furthermore, the performance of the proposed algorithm was evaluated in terms of accuracy and computation complexity. We first compared the performance of our algorithm with that of ICA^17^, POS^35^, and CHROM^36^. We then compared the performances when ICA, POS, and CHROM were each applied to the landmark-based face skin extraction method. We also compared the performance of the landmark-based approach with the rectangle^17^-, bottom^33^- and polygon^34^-face based methods shown in Fig. 4. Furthermore, we validated our proposed algorithm on the testing dataset, BAMI-RPPG (n = 14). For validation, we compared the performance of our algorithm with state-of-art methods in terms of accuracy and computation complexity.

The accuracy of the algorithm was evaluated by calculating the absolute error (AE) of its estimation:

10 $$ AE\left( t \right) = \left| {HR_{est} \left( t \right) - HR_{true} \left( t \right)} \right| $$

where $HR_{true} \left( i \right)$ is the true HR (bpm) in the *i*th window. The overall evaluation of HR estimation was performed on the basis of the absolute value of the AEs (AAE; bpm) and the average of the relative AEs (ARE; %):

11 $$ AAE = \frac{{\mathop \sum \nolimits_{t = 1}^{N} AE\left( t \right)}}{N} $$

12 $$ ARE = \frac{{\mathop \sum \nolimits_{i = 1}^{N} \frac{AE\left( t \right)}{{HR_{true} \left( t \right)}} \times 100}}{N} $$

where N is the total number of windows used for the HR estimation.

To determine the computation complexity, we investigated the computation time for all stages (face detection, face skin extraction, PPG acquisition, and HR estimation), and evaluated whether the proposed process was achievable in real-time. Over the entire process, the robot needs to perform 30 face detections and 30 face skin extractions, one PPG conversion, and one HR calculation within a second. Thus, we defined the processing time within a second (PTOS; ms) as:

13 $$ PTOS\;\left( {ms} \right) = 30 \cdot \left( {T_{fd} + T_{fse} } \right) + T_{rc} + T_{he} $$

where $T_{fd}$, $T_{fse}$, $T_{rc}$ and $T_{he}$ denote the computation time of face detection, face skin extraction, PPG conversion, and HR estimation, respectively.

## Results

### Results using the UBFC-rPPG dataset

**Figure 5:**
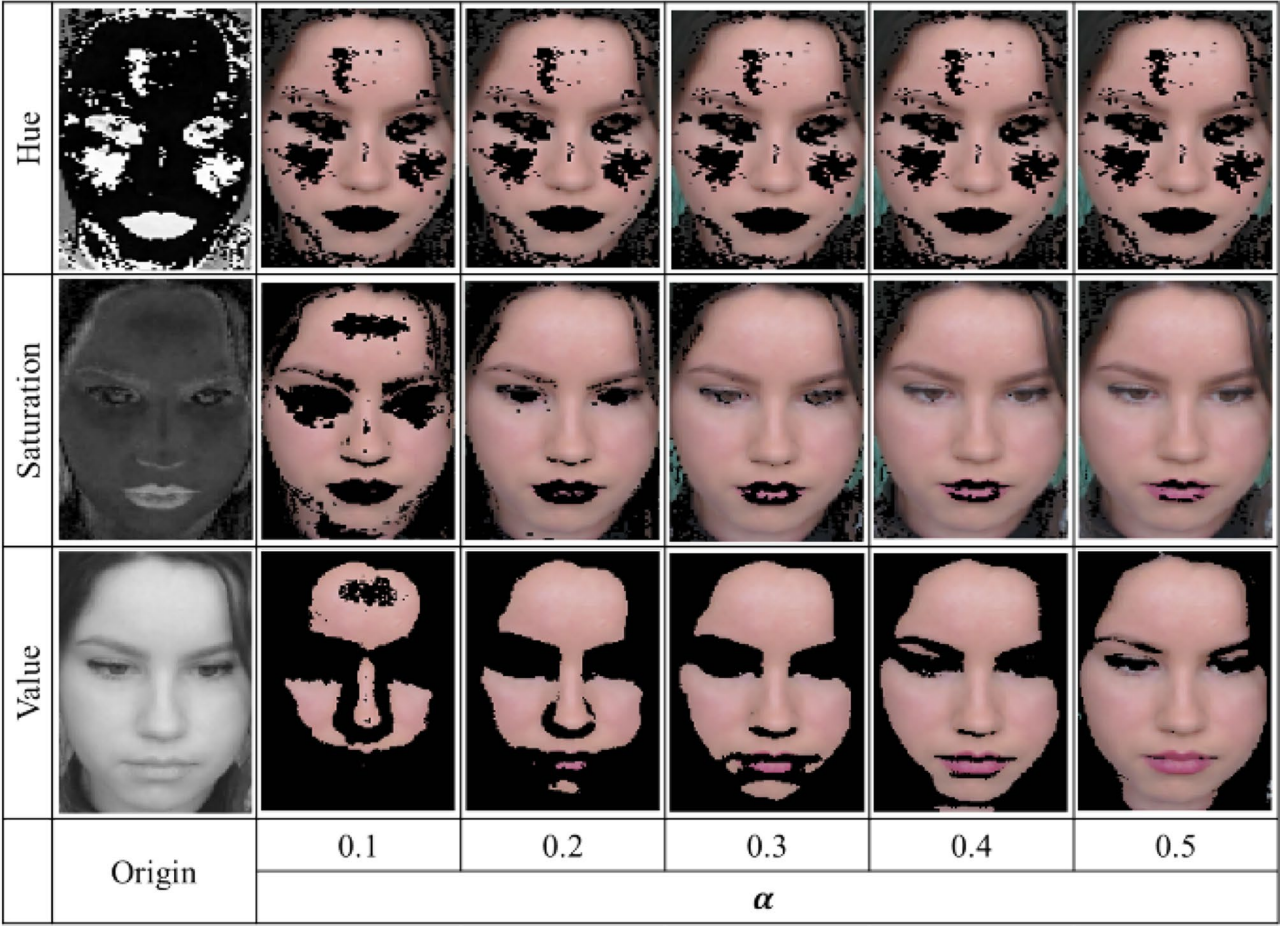
Landmark-based face skin extraction: (**a**) a rectangular face region of interest (ROI)^17^, (**b**) a bottom face ROI^33^, and (**c**) a polygonal face ROI^34^.

Using the UBFC-RPPG dataset, our proposed algorithm was evaluated for a total of 2184 windows, i.e., 42 1 min videos. Figure 5 shows a representative example of the proposed face skin extraction method based on different features (H, S, and V) and different values of α (0.1–0.5). The results show that using the S feature preserves more face skin pixels when α = 0.2. We further investigated the effect of both HSV and RGB features for all 42 subjects, by performing skin segmentation based on different features (H, S, V, R, G and B), and estimated HRs by converting the skin images to a PPG signal. Table 1 summarizes the resultant AAE values according to each feature (H, S, V, R, G, and B) for different values of the parameter α (0.1–0.5). Among all possible features, S provided the lowest AAE, with a value of 0.71 bpm, when α = 0.2.

**Table 1:** AAE values for face skin extraction based on H, S, V, R, G, and B features for different values of the parameter α, using the UBFC-RPPG dataset.

Dataset	$\alpha$	HSV			RGB		
		H	S	V	R	G	B
UBFC-RPPG	0.1	1.94	0.73	1.22	1.23	1.15	1.29
	0.2	1.63	0.71	1.04	0.98	1.07	0.99
	0.3	1.43	0.82	0.87	0.96	0.92	0.94
	0.4	1.46	0.94	0.85	0.93	0.92	0.88
	0.5	2.57	0.96	0.86	0.93	0.89	0.89

Table 2 compares the characteristics of AAE, ARE, and PTOS. When ICA, POS, and CHROM were used without face skin extraction, the AAE values (i.e., the accuracy) were 2.09 bpm, 1.26 bpm, and 1.13 bpm, respectively. When the landmark- based facial skin analysis was applied, the AAE values decreased to 0.79 bpm (i.e., the accuracy was enhanced). However, the computation time, PTOS, significantly increased, to 80,181 ms on an ADM Ryzen 5 3400G CPU at 3.70 GHz personal computer. Conversely, our method provided not only low AAE and ARE values of 0.71 bpm and 0.75%, respectively, but also a low PTOS of 275 ms, which means that our method is achievable in real-time. Notably, a PTOS of 275 ms is approximately 290 times faster than the other landmark-based methods (80,180 ms).

**Table 2:** Comparison of the performances of various methods, in terms of accuracy (AAE and ARE) and computation time (PTOS), on UBFC-RPPG (42 datasets).

Face skin extraction	None			Landmark^41^									Our method
				Rectangle^17^			Bottom face^33^			Polygon face^34^			
PPG conversion	ICA^17^	POS^35^	CH-ROM^36^	ICA^17^	POS^35^	CH-ROM^36^	ICA^17^	POS^35^	CH-ROM^36^	ICA^17^	POS^35^	CH-ROM^36^	
AAE ± Std. (bpm)	2.09 ± 3.65	1.26 ± 2.29	1.13 ± 2.02	2.62 ± 3.31	0.79 ± 1.43	0.78 ± 1.44	0.79 ± 1.43	0.79 ± 1.43	0.79 ± 1.43	1.05 ± 1.87	0.91 ± 1.58	0.80 ± 1.48	0.71 ± 1.38
ARE ± Std. (%)	2.07 ± 3.58	1.28 ± 2.34	1.19 ± 2.12	2.66 ± 3.33	0.82 ± 1.48	0.82 ± 1.52	0.82 ± 1.48	0.82 ± 1.48	0.82 ± 1.48	1.07 ± 1.89	0.94 ± 1.64	0.84 ± 1.54	0.75 ± 1.46
PTOS (ms)	245	244	244	80,181	80,180	80,180	80,181	80,180	80,180	80,181	80,180	80,180	275
CPU Info	AMD Ryzen 5 3400G at 3.70 GHz												

### Results using the BAMI-rPPG dataset

Our proposed algorithm, based on the S feature and with α = 0.2, was applied to the test dataset (BAMI-RPPG). The overall performance for all 14 subjects is summarized in Table 3, where we compare the landmark-based approach to our proposed algorithm, in terms of AAE, ARE and POTS. The results show that our algorithm provides a low AAE of 0.82 bpm and an ARE of 1.12%. The landmark-based approaches yield AAEs ranging between 0.77 and 2.03 bpm, and the AREs ranged between 1.04 and 2.39%. The proposed algorithm showed similar superior accuracy when compared to the landmark-based approach, which required high computation complexity. The POTS of the proposed algorithm was 275 ms, 290 times faster than the landmark-based approaches. This indicates that RSVR can obtain accurate HR estimation in real-time, even when using low-power hardware.

**Table 3:** Performance comparison of various methods in terms of accuracy (AAE and ARE) and computation time (POTS): independent testing dataset – BAMI-RPPG (n = 14).

Subjects	Face skin extraction	Landmark^41^									Our method
		Rectangle ^17^			Bottom face ^33^			Polygon face ^34^			
	PPG acquisition	ICA^17^ AAE (bpm) ARE (%)	POS^35^ AAE (bpm) ARE (%)	CH-ROM^36^ AAE (bpm) ARE (%)	ICA^17^ AAE (bpm) ARE (%)	POS^35^ AAE (bpm) ARE (%)	CH-ROM^36^ AAE (bpm) ARE (%)	ICA^17^ AAE (bpm) ARE (%)	POS^35^ AAE (bpm) ARE (%)	CH-ROM^36^ AAE (bpm) ARE (%)	
1		1.51 (2.11)	1.37 (1.88)	1.45 (1.99)	1.13 (1.55)	1.26 (1.73)	1.40 (1.92)	0.80 (1.15)	0.80 (1.15)	1.22 (1.73)	0.84 (1.21)
2		0.82 (1.28)	1.06 (1.63)	1.01 (1.56)	0.76 (1.19)	0.83 (1.28)	1.03 (1.60)	0.48 (0.75)	0.49 (0.76)	0.54 (0.84)	0.56 (0.86)
3		0.66 (0.87)	0.56 (0.74)	0.65 (0.86)	0.63 (0.83)	0.57 (0.76)	0.65 (0.86)	0.75 (0.99)	0.62 (0.81)	0.66 (0.87)	0.55 (0.73)
4		0.77 (1.09)	0.57 (0.82)	0.61 (0.86)	0.93 (1.32)	0.71 (1.02)	0.79 (1.13)	0.59 (0.84)	0.42 (0.59)	0.47 (0.67)	0.63 (0.89)
5		0.57 (0.81)	0.55 (0.79)	0.57 (0.81)	0.42 (0.59)	0.38 (0.53)	0.39 (0.55)	0.42 (0.60)	0.41 (0.58)	0.45 (0.64)	0.48 (0.69)
6		1.02 (1.09)	1.06 (1.14)	1.42 (1.49)	0.99 (1.05)	0.91 (0.99)	0.95 (1.02)	17.4 (18.7)	0.71 (0.77)	1.03 (1.11)	0.65 (0.70)
7		0.73 (0.84)	0.73 (0.84)	0.67 (0.78)	0.91 (1.01)	0.59 (0.68)	1.13 (1.30)	0.97 (1.12)	1.43 (1.69)	1.70 (1.99)	1.00 (1.18)
8		0.40 (0.54)	0.44 (0.61)	0.44 (0.60)	0.41 (0.56)	0.48 (0.66)	0.47 (0.64)	0.39 (0.53)	0.42 (0.57)	0.46 (0.63)	0.35 (0.48)
9		0.51 (0.67)	0.60 (0.79)	0.71 (0.93)	0.54 (0.70)	0.63 (0.83)	0.68 (0.88)	0.53 (0.70)	0.57 (0.75)	0.60 (0.78)	0.53 (0.70)
10		0.45 (0.58)	0.48 (0.63)	0.66 (0.87)	0.49 (0.64)	0.54 (0.70)	0.51 (0.67)	0.45 (0.58)	0.48 (0.62)	0.49 (0.63)	0.47 (0.61)
11		1.97 (3.13)	1.60 (2.53)	2.04 (3.23)	0.76 (1.18)	0.83 (1.28)	1.22 (1.88)	0.95 (1.47)	0.80 (1.23)	0.79 (1.22)	0.80 (1.24)
12		2.45 (3.75)	2.78 (4.25)	2.53 (3.87)	2.57 (3.88)	2.64 (4.05)	2.65 (4.04)	1.11 (1.72)	1.40 (2.21)	1.04 (1.61)	1.98 (3.04)
13		2.33 (3.07)	0.74 (0.99)	0.60 (0.79)	1.83 (2.40)	1.18 (1.54)	2.39 (3.10)	1.19 (1.55)	0.71 (0.93)	0.89 (1.16)	1.12 (1.45)
14		1.36 (1.67)	1.27 (1.57)	2.00 (2.38)	4.33 (5.35)	1.59 (1.96)	2.20 (2.69)	2.31 (2.81)	1.55 (1.90)	1.94 (2.37)	1.52 (1.85)
Mean		1.11 (1.54)	0.99 (1.37)	1.10 (1.50)	1.19 (1.59)	0.94 (1.29)	1.18 (1.59)	2.03 (2.39)	0.77 (1.04)	0.88 (1.16)	0.82 (1.12)
POTS		80,181	80,180	80,180	80,181	80,180	80,180	80,181	80,180	80,180	275
CPU Info		AMD Ryzen 5 3400G @ 3.70 GHz									

**Figure 6:**
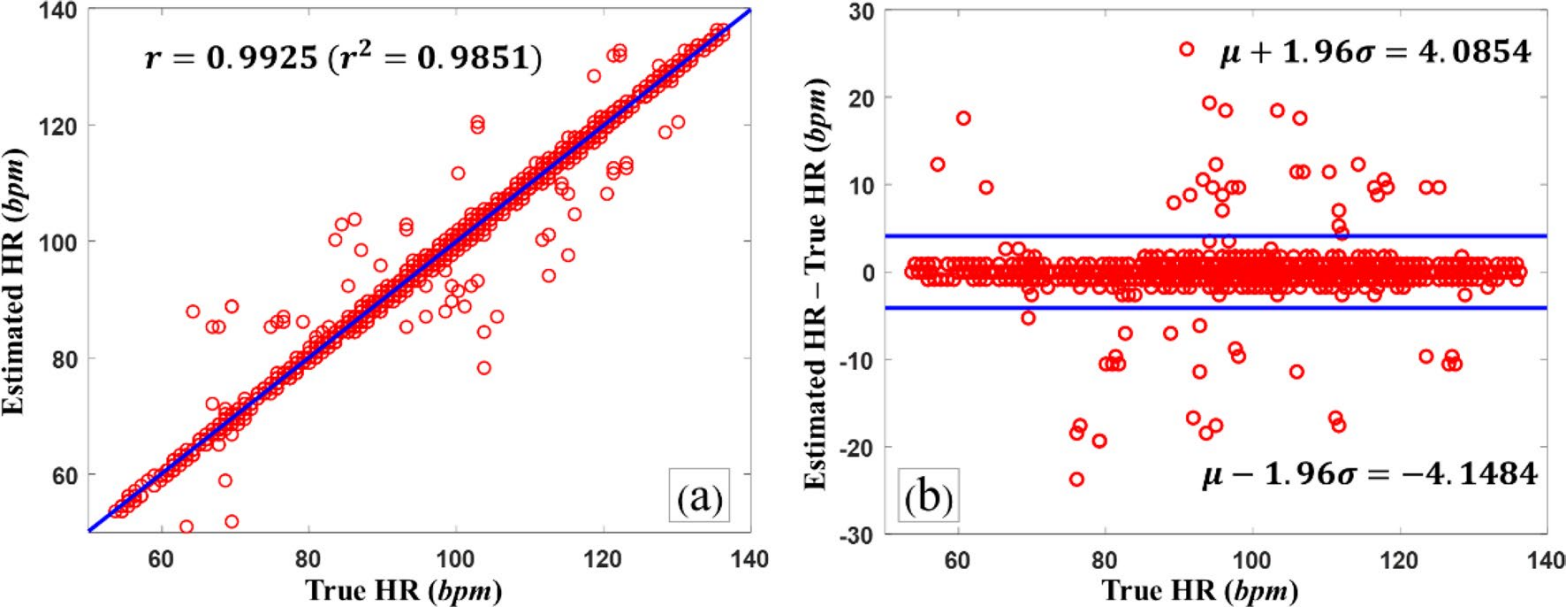
(**a**) Pearson correlations between estimated HRs and true HRs (r = 0.9925). (**b**) Bland–Altman plot (μ = − 0.0315 and σ = 2.1005).

Figure 6a displays the Pearson correlations between the estimated and true HRs. The Pearson correlation coefficient of our model was 0.9925 ($r^{2} = 0.9851$). Figure 6(b) shows a Bland–Altman plot for the estimated and true HRs, with a limit of agreement (LOA) between − 4.05 and 4.09 bpm (mean − 0.0315 bpm, standard deviation 2.1005 bpm).

### Analysis of motion and lighting variation

The PPG acquisition is based on non-contact reflectance; thus, its signal-to-noise (SNR) is relatively low, especially under ambient light changes and movement artifacts. In both of the datasets (UBFC-RPPG and BAMI-RPPG) used in this study, the detected face was relatively immobile, and the ambient light was relatively constant, thus we were able to obtain accurate HR estimation results. If the detected face is in fast motion and/or there is a fast or strong change in the ambient light, the face skin ROI can undergo dynamic changes, which, in turn, results in a low-quality PPG signal and an inaccurate HR estimation.

To investigate the performance of our method under these adverse conditions, we performed additional experiments. We recorded 1-min videos in which the subject was moving their head rapidly, along with further 1-min videos in which the subject was placed under highly variable ambient light conditions. A transmitted PPG signal was obtained using a finger-type oxygen saturation device to record the reference HR. Under these two adverse conditions, our algorithm provided AAEs as high as 2.79 bpm and 4.11 bpm, and the AREs increased by 3.89% and 5.57% (for the high motion and variable ambient light, respectively); this is summarized in Table 4. As a possible solution, we applied the recently introduced finite state machine framework^8^, which automatically eliminates inaccurate estimates based on four states: stable, recovery, alert, and uncertain. Ever second, the FSM framework evaluates its own state based on the estimated results, and also evaluates the signal quality. A stable state indicates that the estimated HR is highly likely to be accurate, and thus is declared valid. A recovery state indicates that the estimated HR is to some extent likely to be accurate, but there is a need to explore a possible transition to a stable state. An alert state indicates that the estimated HR is somewhat likely to be inaccurate, whereas an uncertain state indicates that the estimated HR is highly likely to be inaccurate. The FSM framework transits from one state to another every second in response to the estimation accuracy indicators, namely the crest factor (CF) and the change in HR between consecutive windows. Details of this framework are presented in^8^. The FSM automatically validates the estimation results and ignores inaccurate estimation results, such as those caused by extremely low SNRs in PPG signals.

**Table 4:** HR estimation performance comparison between the proposed method and the FSM method according to the noise type.

Noise Types	Proposed		Proposed + FSM		
	AAE (bpm)	ARE (%)	AAE (bpm)	ARE (%)	VHR (%)
Head movement	2.79 ± 5.17	3.89 ± 7.19	0.56 ± 0.47	0.81 ± 0.67	63.96
Lighting interference	4.11 ± 9.06	5.57 ± 12.19	0.89 ± 1.93	1.26 ± 2.73	72.97

Values are reported as means ± standard deviations.

**Figure 7:**
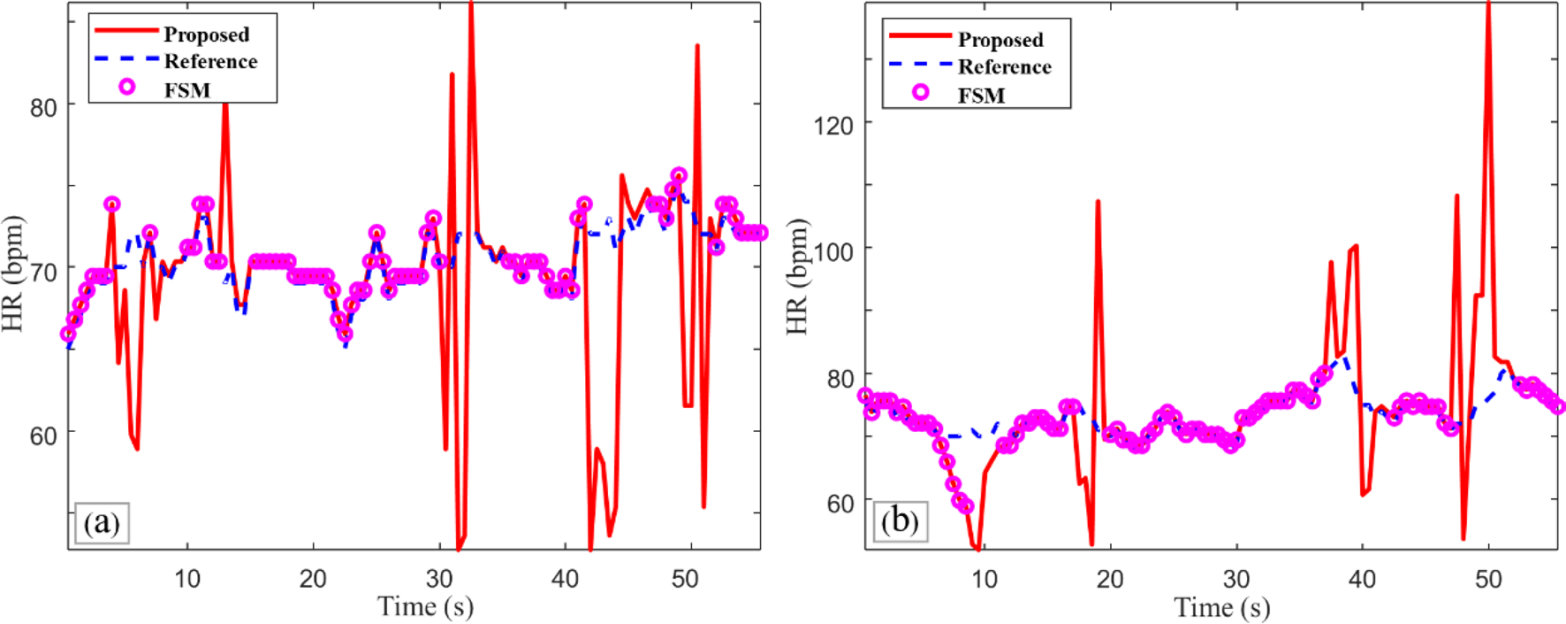
Comparison of the estimated HR results using our proposed method with and without the FSM framework. The HR is estimated using the proposed method, with detected noise removed by the FSM framework: (**a**) under the rapid head movement condition, (**b**) under the changes in ambient lighting condition.

Table 4 shows that, with the FSM framework applied, the AAE values decreased to 0.56 bpm and 0.89 bpm, for the high motion and variable ambient light, respectively. However, the valid HR rate (VHR; %), which is the percentage of valid results among all the windows, were 63.96% and 72.97%, indicating that 36.04% and 27.03% of the estimated results were ignored (or the high motion and variable ambient light, respectively). Figure 7 compares the estimated HR results using our proposed method with and without the FSM framework. However, the FSM framework has a critical drawback in that some estimation results are discarded; hence, the FSM may not provide continuous HR results. This is a limitation, as features such as HR variability cannot be employed.

## Discussion and conclusion

We presented a system for an active and autonomous estimation of HRs using a PPGI mounted on a robotic device. Our proposed system makes it possible to measure HRs during daily life activities without space restrictions, and can be applied in various medical fields. For instance, it can be used for the early detection of heart rate variability-related diseases, such as asymptomatic atrial fibrillation (AF)^48^, by actively monitoring HR variability. Regarding AF, some patients have no symptoms, a condition referred to as asymptomatic AF; these patients may present with devastating thromboembolic consequences or a tachycardia-mediated cardiomyopathy^49^. If a robot can obtain remote HRs or HR variability from a person in daily life or a patient undergoing routine clinical procedure, it can identify undiagnosed AF patients and provide the information to that patient. In addition to AF, we believe that our R-AAH framework provides HR variability analysis without space constraints as the existing PPGI techniques have provided HR variability analysis^50–53^. HR variability is universally accepted as a non-invasive marker of autonomic nervous system activity and can be related to stress and emotional reactions^54–56^. Stress is associated with an increased risk of cardiovascular disease, and vagal tone is considered to be a possible determinant of the stress effects. Emotional response and physiological arousal are adjusted by the central autonomic network, which can be reflected by HR variability. However, in order to achieve such AF detection and HR variability analysis, we should consider the associated challenging issues to accurately perform peak detection from PPG signals with low SNRs. This study is the first step in realizing such active medical services.

To show that our R-AAH framework can be used in real public places, the robot was made to navigate a specific space while avoiding obstacles. In future work, a robot has to minimize irritability by measuring a person who is standing or sitting in public at a distance of a few meters from an immobile person. Such minimal irritability is an essential part of our R-AAH framework because some clients will certainly be irritated, maybe even scared by a robot coming into the patient rooms to measure their HRs. A recent study reported that some of elderly clients perceived the medical robot as nonsense or became irritated while most clients welcomed the robot with curiosity^57^. When we apply our R-AAH framework to the actual medical field, the response level of the patients may differ from culture to culture, so various investigations should be preceded in the future.

**Figure 8:**
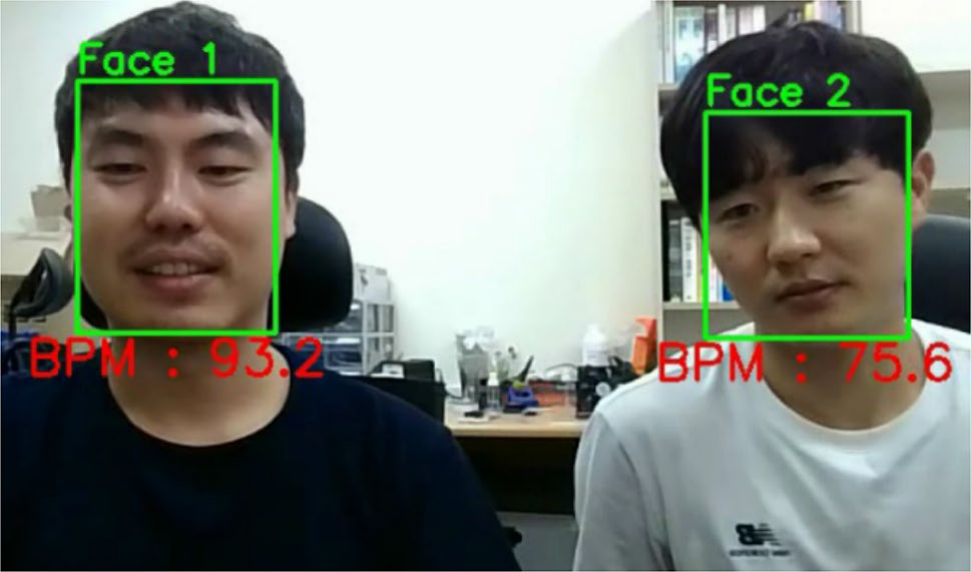
Simultaneous HR estimation of two subjects (additional dataset): An ID is automatically assigned to each face, starting from one, and the face frames from each subject are converted to HR values (both participants provided written informed consent for the publication of identifying images in an online open-access publication).

Throughout this paper, we focused on evaluating the accuracy of the HR estimation, which is one of the most important issues for PPGI. In order for our proposed system to be applied to real-world situations, however, we should also consider more complex environments. We also investigated the performance of the proposed method when there were multiple subjects in the designated space. Similar to the previous simulation, two participants were closely positioned in an indoor environment, and the robot navigated the area with SLAM, searching for a human using face detection. If the two faces were simultaneously detected, the face images were recorded separately, and each facial skin area was extracted with the relative S value range. The CHROM method was then applied for PPG acquisition. Figure 8 shows a video frame from this experiment, where two faces were simultaneously detected, and an estimation of the HR value for each face was provided. The recorded video is available in the supplementary video files. The results show that our proposed system is able to estimate the HRs of multiple subjects at the same time.

The proposed algorithm, combined with the FSM framework, yields a PTOS of 276 ms, which is achievable in real-time. As summarized in Table 5, each PTOS was 243.3 ms for face detection, 30.6 ms for face skin extraction, 0.46 ms for PPG acquisition, 0.72 ms for HR estimation and 1.12 ms for FSM. However, the FSM framework discards some estimation results, which may prevent the acquisition of continuous HR-related physiological information, such as for HRV analysis or atrial fibrillation diagnosis. In future studies, we aim to further investigate how estimations can be improved, without any loss of HR information, even in the presence of fast head movements or under variable ambient light conditions. We have shown that simultaneous HR estimation from multiple subjects is possible. In future work, we aim to continue investigating dynamic issues, such as occlusion or ID assignment with tracking.

**Table 5:** Computation time for each stage of the proposed method with the FSM framework.

Types	Face detection	Proposed method			
		Face skin extraction	PPG acquisition	HR estimation	FSM
Cycle time (ms)	8.11	1.02	0.46	0.72	1.12
Cycle counts during 1 s	30	30	1	1	1
PTOS (ms)	243.3	30.6	0.46	0.72	1.12

To enable the proposed algorithm to be realizable in real-time, we proposed the Relative Saturation Value Range (RSVR), which effectively extracts the facial skin image, enhancing the performance of HR estimation and reducing the computational complexity. The complexity of the computation can be further reduced by incorporating face tracking algorithms, which may allow the face detection process to be performed intermittently, therefore reducing the amount of computations required. In the future, we plan to investigate an optimized tracking algorithm as a strategy that can minimize the cost of the face detection process without loss of accuracy. In addition, face-tracking algorithms could solve the problem of a subject turning their face to prevent the robot from detecting their face. In both of the datasets (UBFC-RPPG and BAMI-RPPG) used in this study, the face detection rate was 100% because most subjects did not move. In future work, we aim to consider more realistic situations, including more severe facial movement during measurement. We believe that the tracking algorithm could replace the FSM framework, which makes HR variability analysis difficult; this would be a significant improvement.

Another issue that should be considered arises when the color of facial skin and hairs are similar; this is a common challenging issue for the facial skin segmentation task. The UBFC-rPPG dataset used in our experiments included subjects of various races; however, many subjects had similar facial skin and/or hair color. Table 2 shows that, even with some subjects having similar facial skin and hair color, the RSVR provided high HR estimation accuracy. Notably, some hairs were incorrectly identified as facial skin as they were a similar color to the facial skin; however, as hairs do not contain any pulsatile information, the segmented hair images do not contribute to the final PPG signal. In addition, the area of hair is significantly smaller than the area of facial skin; hence, the majority of the segmented area is facial skin. In future work, we would like to address the issue of similar face and hair color, and increase HR estimation accuracy by accurately extracting areas that only have facial skin pixels.

The final issue to be considered is whether the average HRs based on an 8 s window can actually detect arrhythmias such as AF. Many algorithms have been developed to detect AF and are based on P-wave detection or HR variability. In PPG signals, HR variability, or inter-beat interval information, is a key feature for the identification of AF. However, even with inter-beat intervals, most algorithms eliminate outliers to filter out premature or ectopic beats^38,58–60^. This outlier elimination also filters out any incorrect inter-beat intervals that originate from a missed or false pulse peak. Conversely, the window-based average HR approach is less sensitive to the issues that stem from premature/ectopic beats or incorrect inter-beat intervals, and Tables 2 and 3 show that the window-based approach provided accurate HR estimation results. We believe that the 8 s sliding window with a 1 s shift is able to detect cardiac arrhythmia, such as atrial fibrillation. In future work, we aim to investigate whether the window-based HR information can actually be applied for the diagnosis of cardiac arrhythmia diseases, such as AF, in clinical practice. This validation will also be performed using our developed R-AAH platform.

## Supplementary Information

Supplementary Video 1.

Supplementary Legends.

Supplementary Table 1.

## Data Availability

All data generated or analyzed during this study are included with this published article.
