# Supplementary material for: Real-time realizable mobile imaging photoplethysmography
Source: Sci Rep. 2022 May 3;12:7141. doi: 10.1038/s41598-022-11265-x (PMC9065061; doi:10.1038/s41598-022-11265-x)
Supplement: Supplementary file 2 — Supplementary Legends. [file 41598_2022_11265_MOESM2_ESM.docx]

### **Supplementary Video**

Simultaneous HR estimation from two subjects: ID is automatically assigned from one, and the face frames from each subject is converted to the instantaneous HR value.

### **Supplementary Tables**

Supplementary Table 1. Face detection rate comparison from each difficulty group: easy, medium and hard in WIDER FACE dataset
