# Supplementary material for: Real-time realizable mobile imaging photoplethysmography
Source: Sci Rep. 2022 May 3;12:7141. doi: 10.1038/s41598-022-11265-x (PMC9065061; doi:10.1038/s41598-022-11265-x)
Supplement: Supplementary file 3 — Supplementary Table 1. [file 41598_2022_11265_MOESM3_ESM.docx]

**Real-Time Realizable Mobile Imaging Photoplethysmography**

Hooseok Lee, M.S^1,†^, Hoon Ko, M.S^1,†^, Heewon Chung, M.S^1,†^,
Yunyoung Nam, Ph.D ^2^, Sangjin Hong, Ph.D ^3^, and Jinseok Lee, Ph.D^1,*^

^1^Department of Biomedical Engineering, Kyung Hee University, Yongin, Republic of Korea
^2^Department of Computer Science and Engineering, Soonchunhyang University, Asan, Republic of Korea
^3^Department of Electrical Engineering, SUNY-Stony Brook University, NY, USA
^*^gonasago@khu.ac.kr
†these authors contributed equally to this work.

Supplementary Table 1. Face detection rate comparision from each difficulty group: easy, medium and hard in WIDER FACE dataset

| Face detection methods | WIDER FACE Dataset (Test set) | | |
| --- | --- | --- | --- |
|  | Easy (mAP) | Medium (mAP) | Hard (mAP) |
| ACF-WIDER ^1^ | 0.695 | 0.588 | 0.290 |
| Two-stage CNN ^2^ | 0.657 | 0.589 | 0.304 |
| Multiscale Cascade CNN ^3^ | 0.711 | 0.636 | 0.400 |
| Faceness-WIDER ^4^ | 0.716 | 0.604 | 0.315 |
| Multitask Cascade CNN ^5^ | 0.851 | 0.820 | 0.607 |
| CMS-RCNN ^6^ | 0.902 | 0.874 | 0.643 |
| **S3FD** | **0.928** | **0.913** | **0.840** |

* We chose S3FD for face detection

### **References**

1 Ohn-Bar, E. & Trivedi, M. M. To boost or not to boost? on the limits of boosted trees for object detection. *2016 23rd International conference on pattern recognition (ICPR)*, 3350-3355 (2016).

2 Yang, S., Luo, P., Loy, C.-C. & Tang, X. Wider face: A face detection benchmark. *IEEE conference on computer vision and pattern recognition*, 5525-5533 (2016).

3 Yang, B., Yan, J., Lei, Z. & Li, S. Z. Aggregate channel features for multi-view face detection. *IEEE International joint conference on biometrics*, 1-8 (2014).

4 Yang, S., Luo, P., Loy, C.-C. & Tang, X. From facial parts responses to face detection: A deep learning approach. *IEEE International conference on computer vision*, 3676-3684 (2015).

5 Zhang, K., Zhang, Z., Li, Z. & Qiao, Y. Joint face detection and alignment using multitask cascaded convolutional networks. *IEEE Signal Processing Letters* **23**, 1499-1503 (2016).

6 Zhu, C., Zheng, Y., Luu, K. & Savvides, M. Cms-rcnn: contextual multi-scale region-based cnn for unconstrained face detection in *Deep learning for biometrics*, 57-79 (Springer, 2017).
